# Antidepressant-like effects of the hydroalcoholic extracts of *Hemerocallis Citrina* and its potential active components

**DOI:** 10.1186/1472-6882-14-326

**Published:** 2014-09-01

**Authors:** Bingjian Du, Xiaoshuang Tang, Fei Liu, Chunyue Zhang, Guanghua Zhao, Fazheng Ren, Xiaojing Leng

**Affiliations:** CAU & ACC Joint-Laboratory of Space Food, College of Food Science & Nutritional Engineering, Key Laboratory of Functional Dairy Science of Beijing and Ministry of Education, Beijing Higher Institution Engineering Research Center of Animal Product, Beijing Dairy Industry Innovation Team, China Agricultural University, No.17 Qinghua East Road, Haidian, Beijing, 100083 People’s Republic of China

**Keywords:** *Hemerocallis citrina*, Antidepressant-like effects, Neurotransmission, Flavonoids

## Abstract

**Background:**

Herbal therapies are potential alternatives and adjuncts for depression treatment. The present study aims to investigate the antidepressant-like effects of hydroalcoholic *Hemerocallis citrina* extracts and its potential neuropharmacological components.

**Methods:**

Hydroalcoholic *H. citrina* extracts were phytochemically analyzed. Behavioral models, including tail suspension tests and open field tests, were performed to evaluate the antidepressant-like effects of the extracts. A possible mechanism was explored by analyzing brain monoamine neurotransmitters. Toxicity and histopathological analyses were performed to determine whether or not the extracts are safe for oral administration.

**Results:**

The antidepressant-like effects of hydroalcoholic *H. citrina* extracts were mainly related to flavonoids, especially rutin and hesperidin. The extract prepared using 75% ethanol (i.e., HCE75) exhibited the highest active flavonoid content and activity. Orally administered 400 mg/kg of HCE75 significantly induced an antidepressant-like effect, whereas the combination of equivalent rutin and hesperidin dosages exhibited the same profiles. Isobologram analysis showed sub-additive antidepressant interactions between rutin and hesperidin. HCE75 (400 mg/kg, p.o.) increased the serotonin and dopamine levels in the central nervous system. Mortality and lesions were not observed upon oral administration of up to 5000 mg/kg HCE75.

**Conclusions:**

The antidepressant-like effects of hydroalcoholic *H. citrina* extracts are mainly related to flavonoids, especially rutin and hesperidin. The serotonergic and dopaminergic systems may have major roles. The active extract is toxicologically safe for oral administration.

## Background

Depression is a chronic, relapsing, and potentially fatal disorder that affects about 20% of the population. Depression has been projected to become the second most common disorder worldwide by 2020 [1]. Antidepressant drugs often have undesirable side effects, such as cholinergic symptoms, withdrawal issues, sexual dysfunction [2], and worsening insomnia [3]. Therefore, developing effective depression therapies with few side effects is essential. Psychotropic plants that exhibit multiple bioactivity and few side effects have gained significant attention as complementary and alternative medicines [4, 5].

Emerging evidence suggests that plant flavonoids mainly exert beneficial effects on the central nervous system (CNS) by protecting neurons against stress-induced injury, suppressing microglia and astrocyte activation and promoting synaptic plasticity, memory, and cognitive function [6, 7]. These properties depend on the chemical structure of the flavonoids. *Schinus molle* L. (Anacardiaceae) and *Hypericum* species are rich in rutin and have been reported to be antidepressants mediated by their interaction with serotonergic, noradrenergic, and dopaminergic systems [8]. The antidepressant-like effect of hesperidin on mice depends on its interaction with serotonergic 5-HT_1A_ receptors [9].

*Hemerocallis citrina* (daylily) is a plant widely grown in East Asia that has antibacterial [10], antioxidant [11], and nitrite-eliminating activities [12]. *H. citrina* has been clinically efficient in relieving depression in patients aged 11 to 80 years [13]. A previous study showed that the ethanol extract of *Hemerocallis fulva* has an antidepressant-like effect, in which rutin is believed to have an important role [14]. The ethanol extract of *H. citrina* has been recently reported to elicit antidepressant-like effects depending on monoaminergic systems [15]. Some researchers have also suggested that such activity of the ethanol extract is at least partly mediated by neurotrophic [16] and inflammation systems [17]. However, the relationships between specific *H. citrina* neuropharmacological activities and its flavonoid components remain uninvestigated. The scientific evaluation of its antidepressant effects are still not convincing.

In this study, the relationships between the *H. citrina* flavonoid composition and its corresponding antidepressant-like activities were determined by performing tail suspension tests, open field tests, and neurochemical analyses in mice. Toxicity and histopathological analyses were also carefully conducted.

## Methods

### Animals

Male imprinting control region mice (25 g to 36 g in weight) were purchased from Vital River Laboratories (Beijing, PR China). The mice were housed in cages and were given unrestricted access to food and water at 25 ± 1°C. Humidity was controlled at 56% ± 3% in a room maintained on a 12 h light/dark cycle (lights on at 8 a.m.). The mice were randomly assigned into specified experimental groups (n = 10 animals per group) and were utilized only once. All animal procedures were conducted in accordance with the animal care and use guidelines of the China Council on Animal Care (Regulations for the Administration of Affairs Concerning Experimental Animals approved by Decree No. 2 of the State Science and Technology Commission on November 14, 1988). The experiments were approved by the Animal Experimental Welfare and Ethical Inspection Committee, The Supervision, Inspection, and Testing Center of Genetically Modified Organisms, Ministry of Agriculture (Beijing, China).

### Plant materials and preparation of extracts

Dried *H. citrina* flowers (7.3% ± 1.2% moisture content; *n* = 6) were purchased from Wal-Mart (Beijing, PR China). A 100 g botanical sample of the flowers was finely powdered and extracted through maceration three times at 25 ± 2°C for 12 h with 1 L portions of deionized water containing different ethanol concentrations (i.e., 0%, 25%, 50%, 75%, and 100%). Ethanol was selected as solvent to ensure food safety. The *H. citrina* extracts (HCEs) were filtered and lyophilized at -60°C for 48 h. Freeze-dried extracts were produced using deionized water containing 0% (HCE0), 25% (HCE25), 50% (HCE50), 75% (HCE75), and 100% ethanol (HCE100). The HCEs were sealed and stored in a freezer before use.

### Drugs and treatments

D-glucose, bovine serum albumin, quercetin, quercitrin, isoquercitrin, rutin, hyperoside, hesperidin, serotonin (5-HT), norepinephrine (NE), and dopamine (DA) were provided by Sigma-Aldrich (St. Louis, MO, USA). Fluoxetine, sodium pentobarbital, and diazepam were purchased from China National Medicines (Beijing, PR China). All chemicals and reagents were analytical grade unless otherwise stated.

All experiments were performed between 13:00 and 17:00. Different groups of mice were used for each test. The HCEs were dissolved in physiological saline with 2% Tween 80; the other drugs were dissolved in physiological saline immediately before use. The reagents were orally administered (p.o.) at the constant volume of 10 ml/kg body weight. The control group mice received appropriate vehicles.

The mice subjected to the experiment for the antidepressant-like effects of HCE received behavioral tests 60 min after they were administered (p.o.) with the vehicle (physiological saline with 2% Tween 80), fluoxetine (20 mg/kg), and HCE (400 mg/kg). The mice were tested 60 min after they were administered (p.o.) with either the vehicle (physiological saline with 2% Tween 80), 20 mg/kg fluoxetine, 0.1, 1, 2, 4, and 8 mg/kg rutin, 0.03, 0.3, 1, 2, and 4 mg/kg hesperidin, 0.01, 0.1, 0.2, 0.4, and 0.8 mg/kg quercetin, and 0.01, 0.1, 0.2, 0.4, and 0.8 mg/kg quercitrin to investigate their antidepressant effects. The mice underwent behavioral tests 0, 0.5, 1, 2, 3, 4, and 8 h after administering (p.o.) with the vehicle (physiological saline with 10% Tween 80), HCE75 (400 mg/kg), standardized flavonoid mixture (8 mg/kg), and (75:21.5, w/w) rutin/hesperidin fixed-ratio combination (8 mg/kg) to investigate the temporal evolutions of the antidepressant-like effects of the treatments. The mice underwent behavioral tests 1 h after administration (p.o.) of the vehicle (physiological saline with 10% Tween 80), fluoxetine (20 mg/kg), HCE75 (20, 200, 400, 800, and 1600 mg/kg), standardized flavonoid mixtures (0.4, 4, 8, 16, and 32 mg/kg), and (75:21.5, w/w) rutin and hesperidin (0.4, 4, 8, 16, and 32 mg/kg) to investigate their antidepressant-like effects.

The mice of the groups that were not subjected to behavioral tests were decapitated and subjected to brain surgery 66 min after administration (p.o.) of the vehicle (physiological saline with 2% Tween 80), fluoxetine (20 mg/kg), and HCE75 (20, 200, 400, 800, and 1600 mg/kg) to investigate the effects of HCE75 on monoamine neurotransmitter levels in prefrontal cortex and hippocampus of mice without or with behavioral tests. The mice of the blank group were not treated, but similarly underwent brain surgery. The mice of the groups exposed to the behavioral tests were decapitated and underwent brain surgery immediately after the animals received the tests 60 min after the administration (p.o.) of the samples.

### Tail suspension test (TST)

TST is a behavioral model widely used to assess antidepressant-like activities by measuring the mobility effects in each test. The immobility behavior displayed in rodents subjected to unavoidable and inescapable stresses during TST reflects behavioral despair, which reflects depression in humans [18]. The test was performed according to previously described methods [19]. In a typical procedure, acoustically and visually isolated mice were suspended 20 cm above the floor using adhesive tape placed approximately 1 cm from the tip of their tails. The total duration of immobility during a 6 min period was recorded. The mice were considered immobile only when they passively hung or stayed completely motionless. The video was analyzed using Smart 3.0.02 (Panlab, USA).

### Open-field test (OFT)

False-positive results can be obtained in TST for agents with psychostimulant effects that could be measured through OFT [20]. The mice were evaluated in an open-field paradigm, as previously described [21]. The mice were individually placed in a Plexiglass box (40 cm × 40 cm × 50 cm) divided into 16 squares. Squares crossed with all paws were considered as indicators of locomotor activity. The behavioral parameters were recorded for 6 min. The floor of the open-field apparatus was cleaned between tests.

### Phytochemical analysis of HCE

The total carbohydrate content was measured by phenol–sulfuric acid methods using D-glucose as a standard [22]. Protein content was measured by Bradford assay, using bovine serum albumin as a standard [23]. The crude fat content was measured using the method 920.39 from the Association of Official Analytical Chemists [24]. The total flavonoid content was assessed through the aluminum chloride colorimetric method and expressed as quercetin equivalents [25]. Flavonoid profile analyses were conducted using a high-performance liquid chromatography (HPLC) system based on previous studies [26]. Qualitative and quantitative analyses were performed using a Purospher STAR RP-18 column (150 mm × 4.6 mm, 5 μm). Solvents A (acetic acid–water, 5:95) and B (pure methanol) were programmed at 0 (95% A + 5% B), 17 (88.5% A + 11.5% B), 28 (88.3% A + 11.7% B), 57 (28.3%A + 71.7% B), and 60 min (95% A + 5% B), at 0.8 ml/min flow rate for chromatography. The column temperature was maintained at 40°C, the injection volume was 10 μl, and the detection wavelength was 254 nm. The flavonoid compounds were identified by comparing the retention time with authentic standards, and quantitative analysis was performed in triplicate to obtain external calibration curves.

### Isobologram analysis

Synergy was graphically assessed through isobologram analysis [27]. In brief, the median effective doses of the drugs and their combinations were determined at a fixed ratio; dose–response curves were plotted in rectangular coordinates. The straight line connecting the points of the drugs represents their theoretical additive combination. The effects of the drugs are purely additive (no interaction) when the point of their combination and their S.E.M. lie on this line. Super-additivity exists when the points lie below this line, but sub-additivity (antagonism) exists when the points lie above this line.

### Monoamine neurotransmitter levels

The 5-HT, NA, and DA concentrations in the mouse brain were measured through HPLC with electrochemical detection, as previously described [28]. The mice were decapitated, and their brains were immediately removed. The frontal cortex and hippocampus were carefully dissected and stored at -80°C until measurement (no more than 1 week). The brain tissues were homogenized in an ice-cold 0.4 M perchloric acid (5 μl/mg) solution that contains 5 mM sodium bisulfate and 0.04 mM EDTA to avoid oxidation. The homogenate was centrifuged at 15,000 × *g* for 15 min at 4°C. Approximately 10 μl of the resulting supernatant was chromatographed on a Purospher STAR RP-18 column (150 mm × 4.6 mm, 5 μm). Separation was performed in an isocratic elution mode using a mobile phase at 30°C column temperature. The phase comprised 8% methanol and 92% water, containing EDTA (0.5 mM), triethylamine (5 mM), sodium 1-heptanesulfonate (0.5 mM), Na_2_HPO_4_°12H_2_O (20 mM), and citrate (50 mM), at 1 ml/min flow rate. The neurotransmitters were measured using the electrode potential of a glassy carbon working electrode at +650 mV against the Ag/AgCl electrode. The 5-HT, NA, and DA were identified and quantified by comparing their retention times and peak areas with those of standard solutions; their contents were expressed as ng/g wet weight tissue.

### Acute toxicity and histopathological analysis

Orally acute HCE toxicity was estimated according to the procedure reported by Lorke [29]. Each of the three different animal groups (n = 3 animals per group) were administered (p.o.) with 10, 100, and 1000 mg/kg of extract during the first phase. The doses were then increased to 1600, 2900, and 5000 mg/kg in another three different groups (n = 3 animals per group) when mortality was not observed during the first day of the first phase and 1 week thereafter. The mice were observed for 14 d to note possible mortality.

Histopathological analysis was conducted in a separate series of experiments. Mice (n = 10 animals per group) were orally treated with 5000 mg/kg HCE75 once a day for 1 d or 21 consecutive days, and the mice were sacrificed for histopathological analyses according to the previously described methods [30]. Livers, kidneys, and spleens were immediately collected and placed in formalin. The samples were transferred to a cassette and immersed in multiple baths containing progressively higher volumes of ethanol to dehydrate the tissue, followed by xylene and hot paraffin. More paraffin was added during embedding to create a paraffin block, which allowed the sectioning of the tissues into 2 μm slices. The microtome slices were stained with hematoxylin–eosin stain.

### Statistical analysis

All values were expressed as mean ± S.E.M. The data were analyzed using ANOVA, followed by post hoc Student–Newman–Keuls test for multiple comparisons. Statistically significant differences were denoted by *p* < 0.05. Linear regression was performed along with the ordinary least squares estimation.

## Results and discussion

### Phytochemical profile analysis of HCE

The HCE0, HCE25, HCE50, HCE75, and HCE100 extraction yields and their main compositions are shown in Table 1. Increasing ethanol concentration in the extracting solvents decreased the carbohydrate and protein contents and increased the crude fat and flavonoid contents in the extract. The increase in crude fat content in HCE100 indicates the enhanced fat-soluble compound extraction by pure ethanol. Nevertheless, the extracted fat-soluble compounds prevent the further extraction of the flavonoids [31]. The extracted flavonoid in HCE100 did not rapidly increase.

**Table 1:** **Chemical composition of HCE (%, w/w)**

Sample	Yield	Carbohydrate	Protein	Crude fat	Flavonoid
HCE0	18.2 ± 1.2	80.1 ± 4.1	9.2 ± 0.6	0.3 ± 0.1	0.4 ± 0.1
HCE25	16.9 ± 0.9	81.8 ± 3.3	8.5 ± 0.4	1.1 ± 0.2	0.6 ± 0.1
HCE50	14.9 ± 0.8	75.2 ± 2.9	8.1 ± 1.0	1.7 ± 0.3	0.7 ± 0.1
HCE75	9.2 ± 0.6	69.4 ± 5.3	6.7 ± 0.7	6.3 ± 0.7	2.0 ± 0.2
HCE100	4.4 ± 0.5	11.4 ± 0.8	0.2 ± 0.1	74.1 ± 2.1	2.3 ± 0.2

Data are expressed as mean ± S.E.M. (*n* = 3).

**Figure 1:**
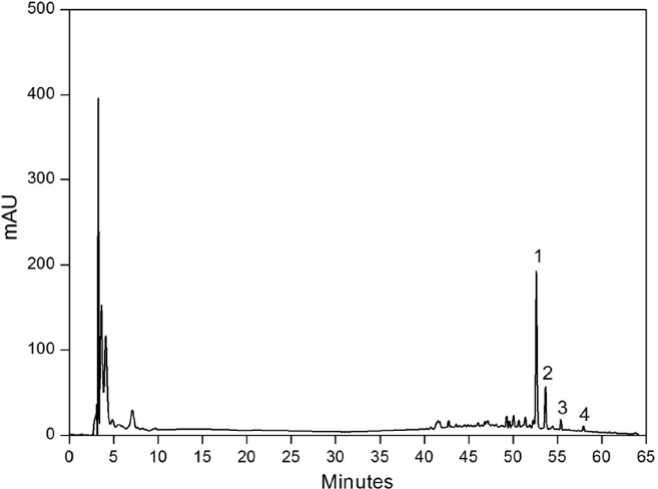
**Flavonoid fingerprint of HCE75 as a representative HPLC chromatogram. The peaks correspond to (1) rutin, (2) hesperidin, (3) quercitrin, and (4) quercetin.**

The flavonoid components of HCE were analyzed through HPLC. Figure 1 illustrates the representative chromatogram of HCE. Peaks 1, 2, 3, and 4 correspond to rutin (52.6 min), hesperidin (53.2 min), quercitrin (54.8 min), and quercetin (57.9 min), respectively. Rutin is the major component of HCE, followed by hesperidin. The quercitrin and quercetin contents were relatively minor. Table 2 lists the main flavonoid components.

**Table 2:** **Composition of the HCE flavonoids (%, w/w)**

Sample	Quercetin	Quercitrin	Isoquercitrin	Rutin	Hyperoside	Hesperidin
HCE0	0.8 ± 0.1	0.6 ± 0.1	ND	96.2 ± 5.7	ND	0.2 ± 0.0
HCE25	5.9 ± 1.0	3.8 ± 0.2	ND	73.2 ± 1.9	ND	15.2 ± 2.1
HCE50	3.7 ± 0.9	1.3 ± 0.4	ND	65.7 ± 8.4	ND	28.1 ± 5.3
HCE75	1.5 ± 0.4	2.1 ± 0.3	ND	74.2 ± 6.8	ND	21.3 ± 3.7
HCE100	3.3 ± 0.7	3.7 ± 0.5	ND	69.2 ± 5.9	ND	24.1 ± 2.4

Data are expressed as mean ± S.E.M. (*n* = 3); ND, not determined.

### Antidepressant-like effects of HCE and neuropharmacological component analysis

**Figure 2:**
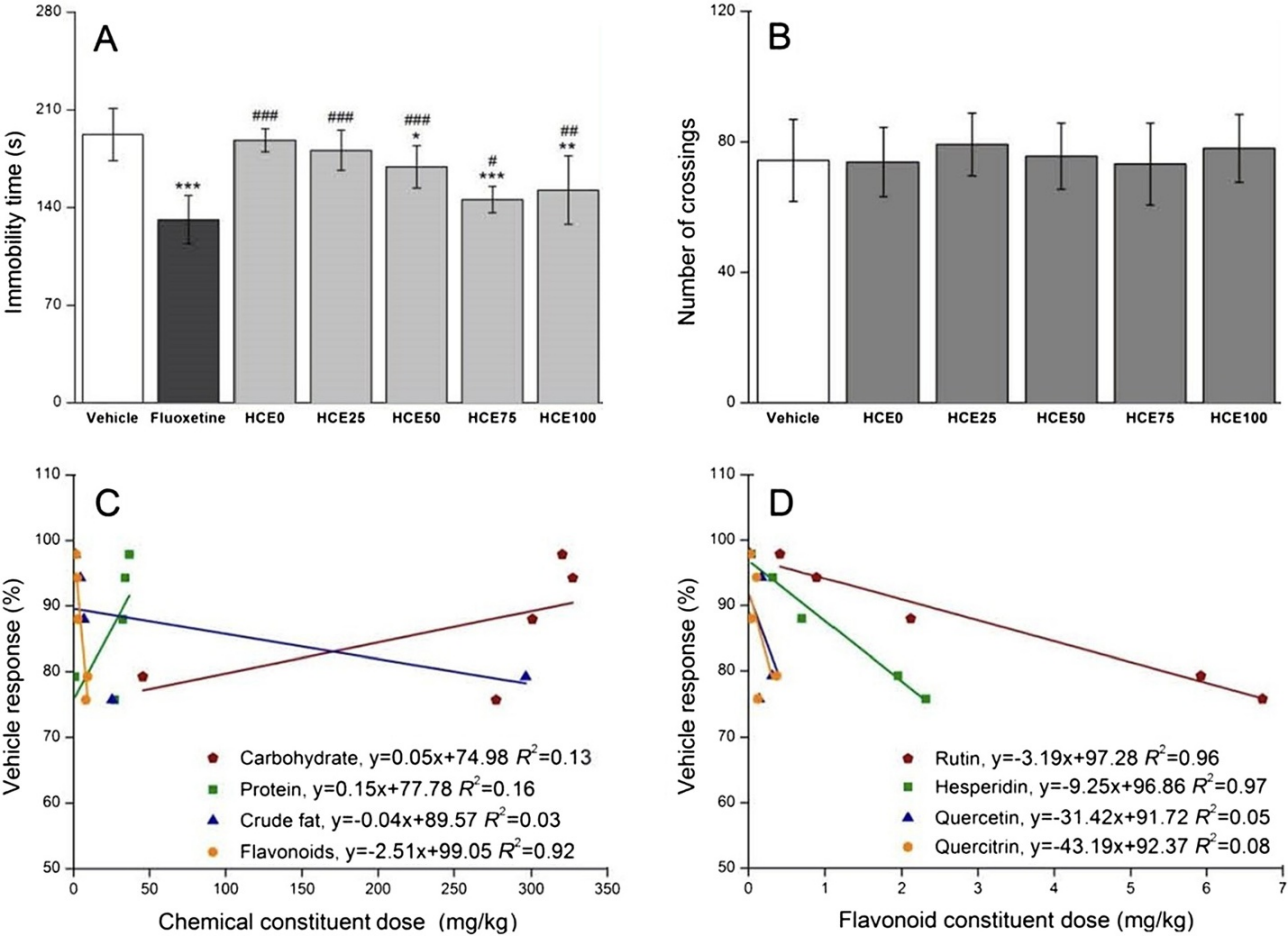
**Effects of HCE on immobility time in TST (A) and line crossings in OFT (B).** The apparent correlations between (C) chemical and (D) flavonoid constituent dosages and the immobility time in HCE in the TST. The results are expressed as% immobility time relative to the control group (vehicle) for the antidepressant-like effects. The mice were tested 60 min after they were administered (p.o.) with the vehicle (physiological saline with 2% Tween 80), fluoxetine (20 mg/kg), and HCE (400 mg/kg). Data are expressed as mean ± S.E.M. (*n* = 10). Data were analyzed using one-way ANOVA for multiple comparisons, followed by post hoc Student–Newman–Keuls test. *p < 0.05, **p < 0.01, and ***p < 0.001 compared with the control group (vehicle); and #*p* < 0.05, ##*p* < 0.01, and ###*p* < 0.001 compared with the positive reference group (fluoxetine). Linear regression was performed along with the ordinary least squares estimation.

The effects of HCE treatment (400 mg/kg, p.o.) on immobility time in TST are presented in Figure 2A. HCE50 (*p* < 0.05), HCE75 (*p* < 0.001), and HCE100 (*p* < 0.01) significantly decrease immobility time compared with the control group, indicating good antidepressant-like performances. The reducing abilities of HCE75 were closest among the three to that of fluoxetine, which is the most common clinical therapy drug used as positive reference. Significant variations in OFT line crossings were not found in Figure 2B, indicating that HCE did not stimulate locomotor activity, thus the antidepressant-like effects of HCE in the TST were specific. The poor anti-immobility correlations with the carbohydrates (*R*^2^ = 0.16), proteins (*R*^2^ = 0.13), and crude fat (*R*^2^ = 0.03), unlike flavonoids (*R*^2^ = 0.92), derived from their dose–response regression analyses shown in Figure 2C indicate a possible disconnect with any antidepressant-like effect [32]. Quercetin (*R*^2^ = 0.05) and quercitrin (*R*^2^ = 0.08) were also ruled out in the regression analyses because of the apparent noncorrelation between the flavonoids and antidepressant-like performance (Figure 2D). By contrast, the high degree of correlation between immobility time and dose indicates that the antidepressant-like effects are closely associated with rutin (*R*^2^ = 0.96) and hesperidin (*R*^2^ = 0.97).

**Figure 3:**
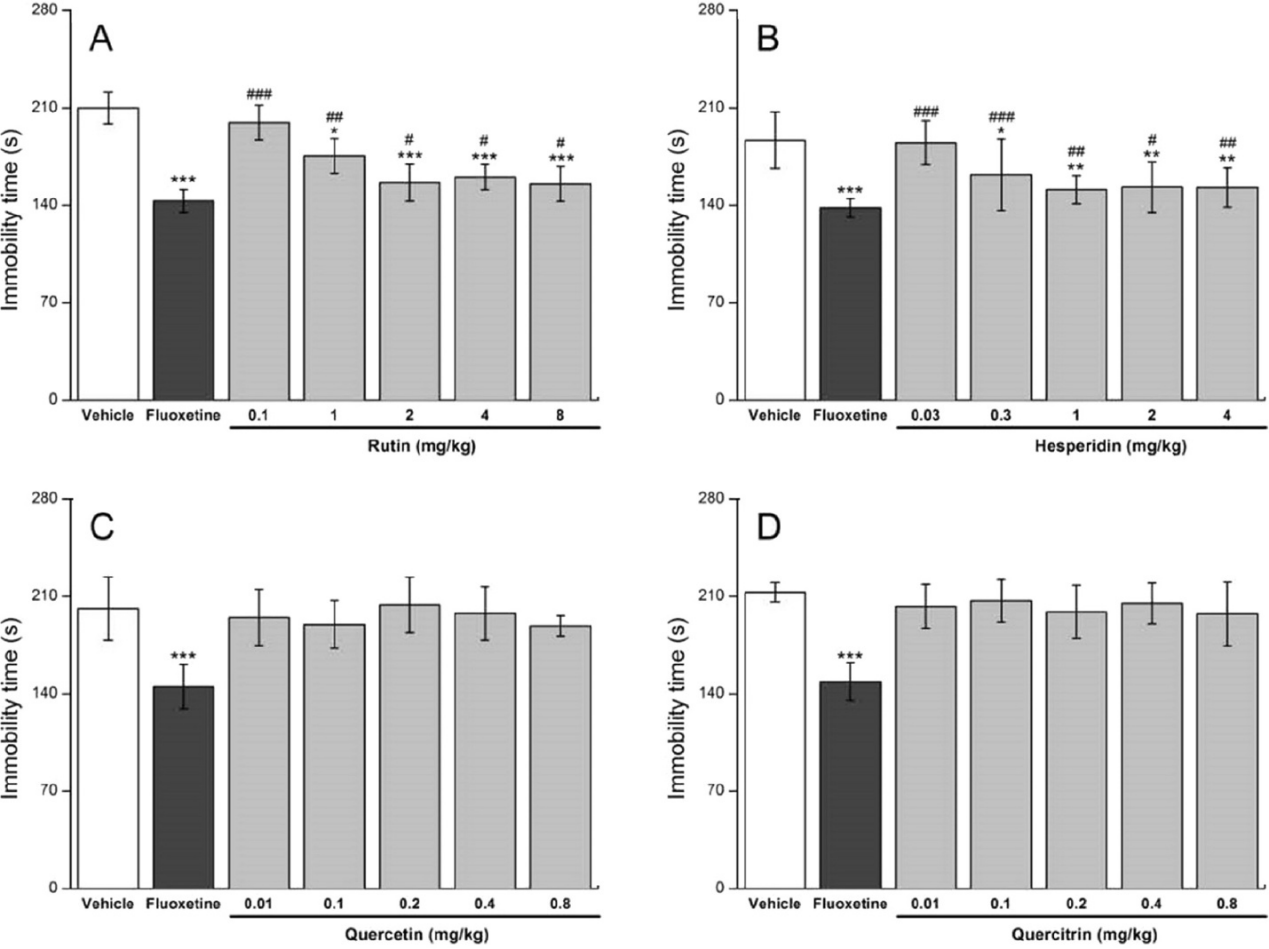
**Effects of the pure rutin (A), hesperidin (B), quercetin (C), and quercitrin (D) on immobility time in TST.** The mice were tested 60 min after they were administered (p.o.) with the vehicle (physiological saline with 2% Tween 80), 20 mg/kg fluoxetine, 0.1, 1, 2, 4, and 8 mg/kg rutin, 0.03, 0.3, 1, 2, and 4 mg/kg hesperidin, 0.01, 0.1, 0.2, 0.4, and 0.8 mg/kg quercetin, and 0.01, 0.1, 0.2, 0.4, and 0.8 mg/kg quercitrin. Data are expressed as mean ± S.E.M. (*n* = 10). Data were analyzed using one-way ANOVA for multiple comparisons, followed by post hoc Student–Newman–Keuls test. *p < 0.05, **p < 0.01, and ***p < 0.001 compared with the control group (vehicle); #*p* < 0.05, ##*p* < 0.01, and ###*p* < 0.001 compared with the positive reference group (fluoxetine).

Pure flavonoids were tested to confirm the hypothesis. Figure 3A and 3B show that pure rutin (2–8 mg/kg) and hesperidin (1–4 mg/kg) elicits significant antidepressant-like actions on mice in TST, similar to that evoked by the equivalent HCE50 (2.1 and 0.7 mg/kg rutin and hesperidin, respectively), HCE75 (6.7 and 2.3 mg/kg rutin and hesperidin, respectively), and HCE100 (5.9 and 1.9 mg/kg rutin and hesperidin, respectively). The orally administered quercetin and quercitrin doses did not exhibit antidepressant-like effects in the present conditions (Figure 3C and 3D). Traces of contributions from carbohydrates, proteins, and crude fat were not observed. Psychostimulant effects were likewise not observed in the OFT of the flavonoids (data not shown). Dimpfel [33] found that 5 to 80 mg/kg rutin doses produced electro-pharmacograms similar to those of clinical antidepressants in rats. A single i.p. hesperidin administration yielded antidepressant-like effects [9].

### Antidepressant-like effect of HCE75 and active flavonoid involvement

**Figure 4:**
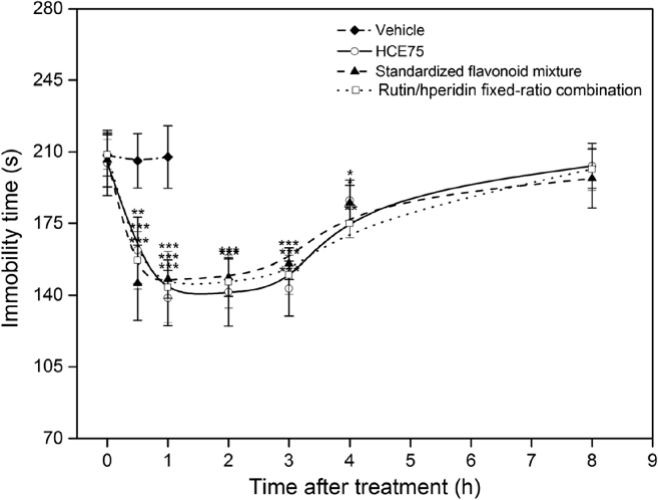
**Temporal evolutions of the antidepressant-like effects caused by HCE75, the standardized flavonoid mixture containing equivalent doses of rutin, hesperidin, quercetin, and quercitrin and the fixed-ratio combination of rutin and hesperidin (75:21.5, w/w) on mice in TST.** The mice were tested 0, 0.5, 1, 2, 3, 4, and 8 h after administering (p.o.) with the vehicle (physiological saline with 10% Tween 80), HCE75 (400 mg/kg), standardized flavonoid mixture (8 mg/kg), and rutin/hesperidin fixed-ratio combination (8 mg/kg). Data are expressed as mean ± S.E.M. (*n* = 10). Data were analyzed using one-way ANOVA for multiple comparisons, followed by post hoc Student–Newman–Keuls test. *p < 0.05, **p < 0.01, and ***p < 0.001 compared with the control group (tested 1 h after vehicle treatment).

The previous analysis reveals that HCE75 is the most active *H. citrina* extract. The temporal behavior of the antidepressant-like effects and the dose-dependent properties (100 mg/kg to 1600 mg/kg) of the extract was estimated. The standardized flavonoid mixture prepared using four types of pure flavonoids and the combination with pure rutin and hesperidin at a fixed ratio (75:21.5, w/w) with respect to the real flavonoid composition of HCE75 (75% rutin, 21.5% hesperidin, 2% quercetin, and 1.5% quercitrin, w/w) were compared. Figure 4 shows the comparison of the three samples. The antidepressant-like effects of HCE75, standardized flavonoid mixture, and rutin/hesperidin fixed-ratio combination reached their maxima (*p <* 0.001) at 1, 0.5, and 0.5 h, respectively. The anti-depressant effects remained significant (*p <* 0.01) up to 4 h after p.o. administration. The temporal evolution curves were consistent. Psychostimulant effects were not observed in OFT (data not shown). Therefore, the functionality of the major antidepressants in HCE75 is mediated by rutin and hesperidin, whereas the contributions of quercetin and quercitrin are insignificant.

**Figure 5:**
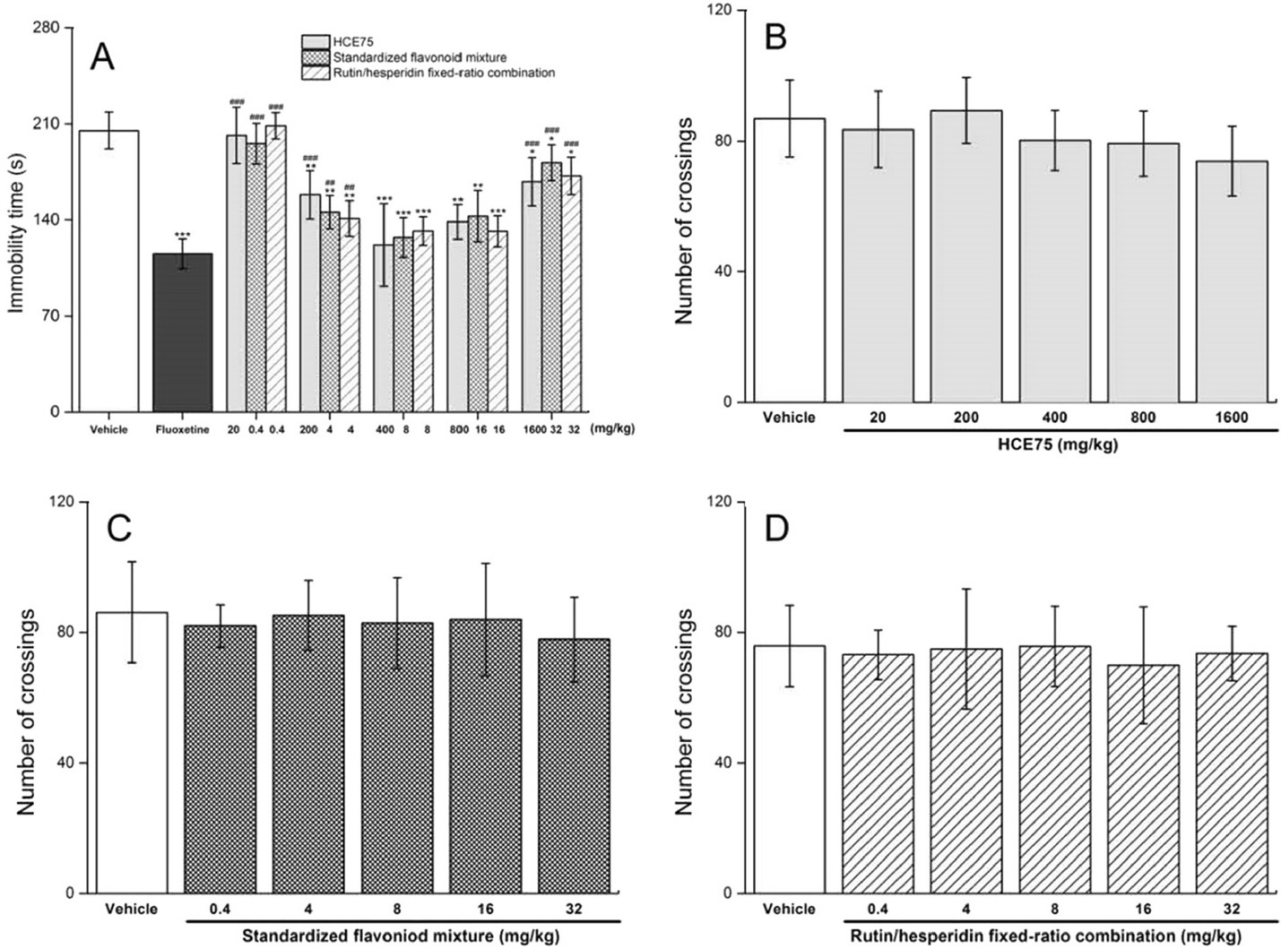
**Effects of HCE75, the standardized flavonoid mixture containing equivalent rutin, hesperidin, quercetin, and quercitrin doses and the fixed-ratio combination of rutin and hesperidin (75:21.5, w/w), on (A) immobility time in TST and (B to D) their respective line crossings in OFT.** The mice were tested 1 h after administration (p.o.) of the vehicle (physiological saline with 10% Tween 80), fluoxetine (20 mg/kg), HCE75 (20, 200, 400, 800, and 1600 mg/kg), standardized flavonoid mixtures (0.4, 4, 8, 16, and 32 mg/kg), and the fixed-ratio rutin and hesperidin combination (0.4, 4, 8, 16, and 32 mg/kg). Data are expressed as mean ± S.E.M. (*n* = 10). Data were analyzed using one-way ANOVA for multiple comparisons, followed by post hoc Student–Newman–Keuls test. *p < 0.05, **p < 0.01, and ***p < 0.001 compared with the control group (vehicle); ##*p* < 0.01 and ###*p* < 0.001 compared with the positive reference group (fluoxetine).

HCE75 (200–800 mg/kg) exhibited a significant anti-immobility effect (*p* < 0.01) in TST compared with the control groups (Figure 5A). Doses more than 800 mg/kg increased immobility time and formed a U-shaped dose–response profile in antidepressant-like action. Insignificant variations in the OFT line crossings (Figure 5B) ruled out the psychostimulant and sedative effects. The phenomenon was often observed during rodent model screening (i.p. or p.o.) on potential antidepressants, which was attributed to either the activation of different pathways at diverse doses [34] or the suppression of the maximum response in the presence of antagonists through non-competitive antagonism [4]. The dose-dependent diversity of the mechanisms of the neuropharmacological effects should be studied further because the highest HCE75 dosage (i.e., 1600 mg/kg) for oral treatment resulted in a hypnotic response (data not shown). The response might negate the antidepressant-like effects.The standardized flavonoid mixture containing equivalent rutin, hesperidin, quercetin, and quercitrin doses and the fixed-ratio combination of rutin and hesperidin induced the same characteristics and changes without significantly changing the OFT line crossings (Figure 5C and 5D). These results indicate that rutin and hesperidin cause the antidepressant effects of HCE75. The contributions of quercetin and quercitrin in the effects were insignificant.

**Figure 6:**
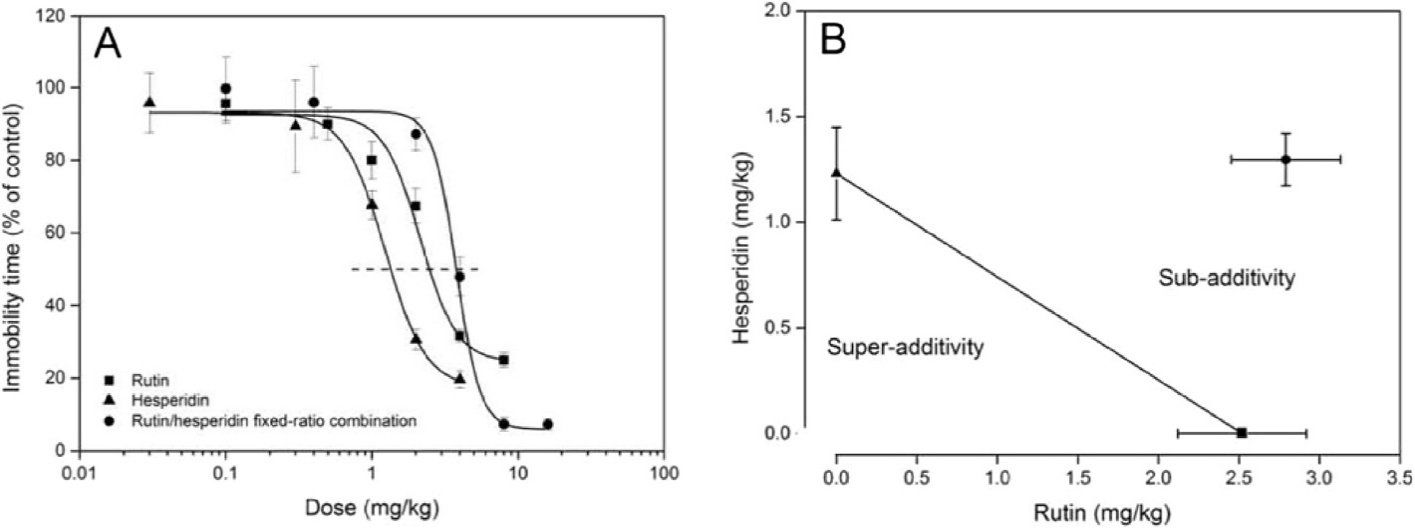
**Dose–response curves of the immobility time in TST with respect to the control groups. (A)** The positive reference group (fluoxetine, 20 mg/kg, p.o.) response was 0 for the antidepressant-like effects of rutin, hesperidin, and the fixed-ratio combination of rutin/hesperidin (75:21.5, w/w). **(B)** The consequent isobolograms. Data are expressed as mean ± S.E.M. (*n* = 10).

Dose–response curves were obtained for rutin, hesperidin, and their fixed-ratio combination (75:21.5, w/w; Figure 6A) to assess the interaction between rutin and hesperidin in the antidepressant-like effects in TST. The data were fitted to logistic dose–response curves via nonlinear regression. Dose-dependent antidepressant-like effects were observed for the chosen dose ranges with 2.5 mg/kg rutin, 1.2 mg/kg hesperidin, and 3.7 mg/kg fixed-ratio rutin/hesperidin combination median effective dose values. The isobolograms obtained for the rutin/hesperidin fixed-ratio combination (Figure 6B) reveal a sub-additive effect. The result implies an antagonistic interaction between rutin and hesperidin. Few studies focused on the assessment of the neuroactive properties of flavonoid-flavonoid interactions. The active potency of a given flavonoid is closely linked to its structure, such as the ortho-dihydroxy structure in the B-ring, the 2-3-double bond in conjugation with a 4-oxo function, and the presence of the 3- and 5-OH functions [35]. More detailed studies are required to elucidate the mechanisms involved in the neuropharmacological interactions.

### Effects of HCE75 on monoamine neurotransmitter levels in mice brains

**Figure 7:**
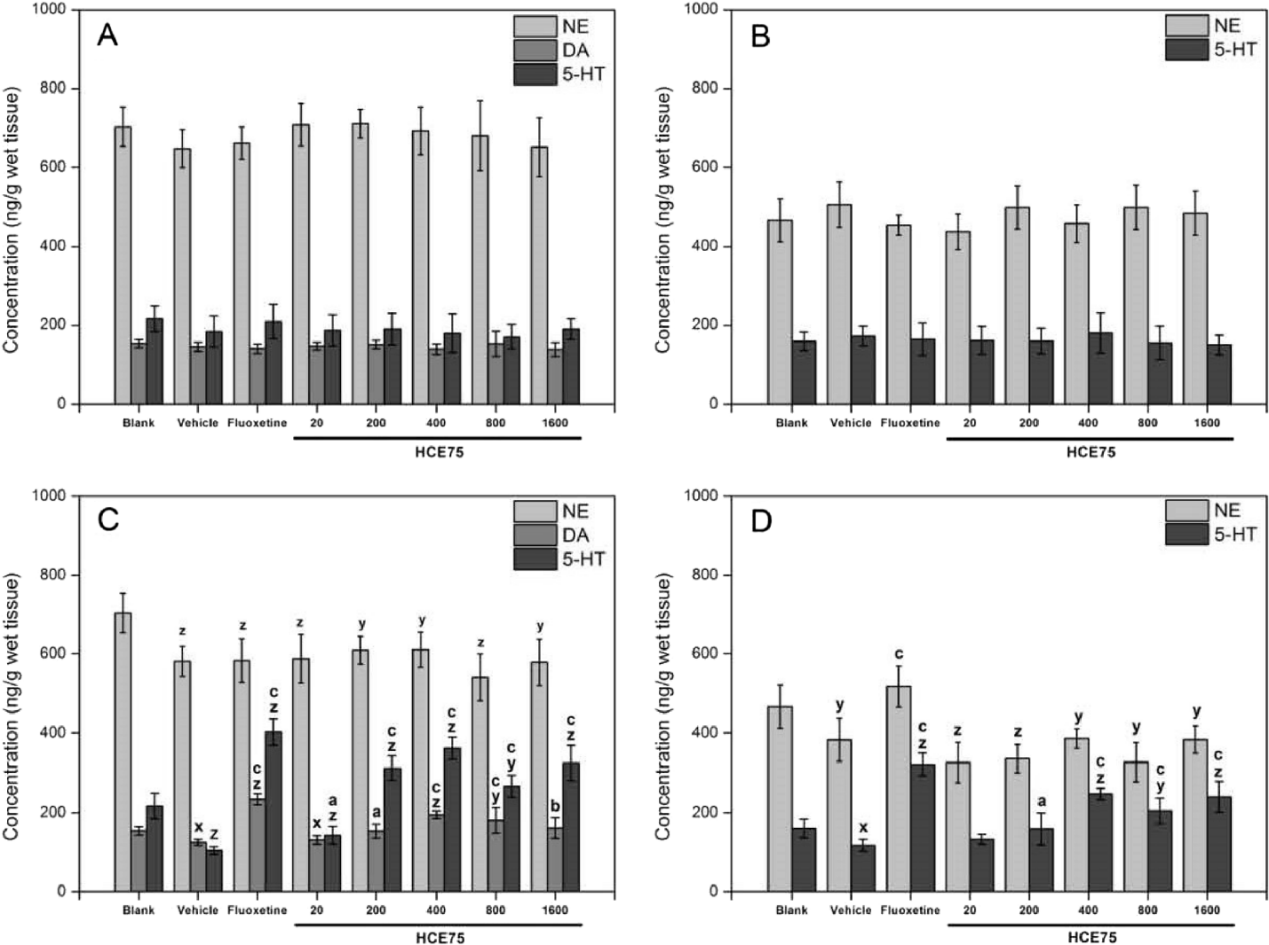
**Effects of HCE75 on monoamine neurotransmitter levels in the prefrontal cortex (A and C) and hippocampus (B and D) of mice without (A and B) or with TST (C and D).** The mice of the groups unexposed to TST were decapitated and were subjected to brain surgery 66 min after administration (p.o.) of the vehicle (physiological saline with 2% Tween 80), fluoxetine (20 mg/kg), and HCE75 (20, 200, 400, 800, and 1600 mg/kg). The mice of the blank group were untreated, but similarly underwent brain surgery. The mice of the groups exposed to TST were decapitated and were subjected to brain surgery immediately after the animals were tested 60 min after treatment. Data are expressed as mean ± S.E.M. (*n* = 10). Data were analyzed using one-way ANOVA for multiple comparisons, followed by post hoc Student–Newman–Keuls test. ^x^ *p* < 0.05, ^y^ *p* < 0.01, and ^z^ *p* < 0.001 compared with the blank group; ^a^ *p* < 0.05, ^b^ *p* < 0.01, and ^c^ *p* < 0.001 compared with the vehicle groups exposed to TST.

Depression symptoms are associated with changes in monoamine neurotransmitter (i.e., NE, DA, and 5-HT) levels in the CNS [1, 36]. The prefrontal cortex and hippocampus, which regulate emotion, motivation, learning, and memory, are essential in depression [37]. Nutt [36] revealed that NE is related to alertness, energy, and attention, and that DA is linked to pleasure, reward, and motivation in life. The 5-HT transmitter is related to compulsion, obsession, and anxiety.Figure 7 shows the monoamine neurotransmitter levels (i.e., NE, DA, and 5-HT) in the prefrontal cortex (Figure 7A and 7C) and hippocampus (Figure 7B and 7D) of the mice without (Figure 7A and 7B) or with TST (Figure 7C and 7D), respectively, after treatment. The DA level in the hippocampus was too low to be detected.

Insignificant differences were observed in the monoamine neurotransmitter levels in the prefrontal cortex (Figure 7A) or hippocampus (Figure 7B) of the blank, vehicle, fluoxetine, and HCE75 treatment groups without TST. The NE levels of HCE75-treated groups with TST were almost similar to that of the vehicle, and the levels significantly (*p* < 0.05) decreased after exposure to the behavioral models. Anti-reduction was observed in the fluoxetine-treated group (Figure 7D). DA and 5-HT levels were significantly higher (*p* < 0.05) with TST and the HCE75 dose of more than 200 mg/kg than the vehicle groups. These levels reached their maxima at 400 mg/kg in almost all cases, and were maintained for doses up to 1600 mg/kg, the highest dose tested in the present work. This point is consistent with the anti-immobility effects of HCE75 described in the previous section. This condition indicates that increasing the 5-HT and DA levels in the CNS is an important parameter in the mechanism of the antidepressant-like response of HCE75. Machado et al. [8] proved the involvement of 5-HT, DA, and/or NE systems in the antidepressant-like action of rutin. The antidepressant-like performance of hesperidin was determined to be related to the 5-HT system, whereas NE and DA systems were indirectly involved [9], consistent with the findings in this study. However, the potential interaction between rutin and hesperidin requires further study. The U-shaped dose–response profile observed in the previous section should not be due to the decrease in antidepressant effect of HCE75, because the increasing effects of HCE75 at doses ranging from 400 mg/kg to 1600 mg/kg on the monoamine neurotransmitter levels in the TST were basically stable.

### Toxicity and histopathological analysis

Plants rich in neuroactive flavonoids are usually inedible, which makes the consideration of safety and tolerability a requirement in plant supplement development. The toxicity study on HCE75 by Lorke [29] revealed that the median lethal dose is not feasibly estimated because mortality was not recorded from the oral administration of 5000 mg/kg HCE75.

**Figure 8:**
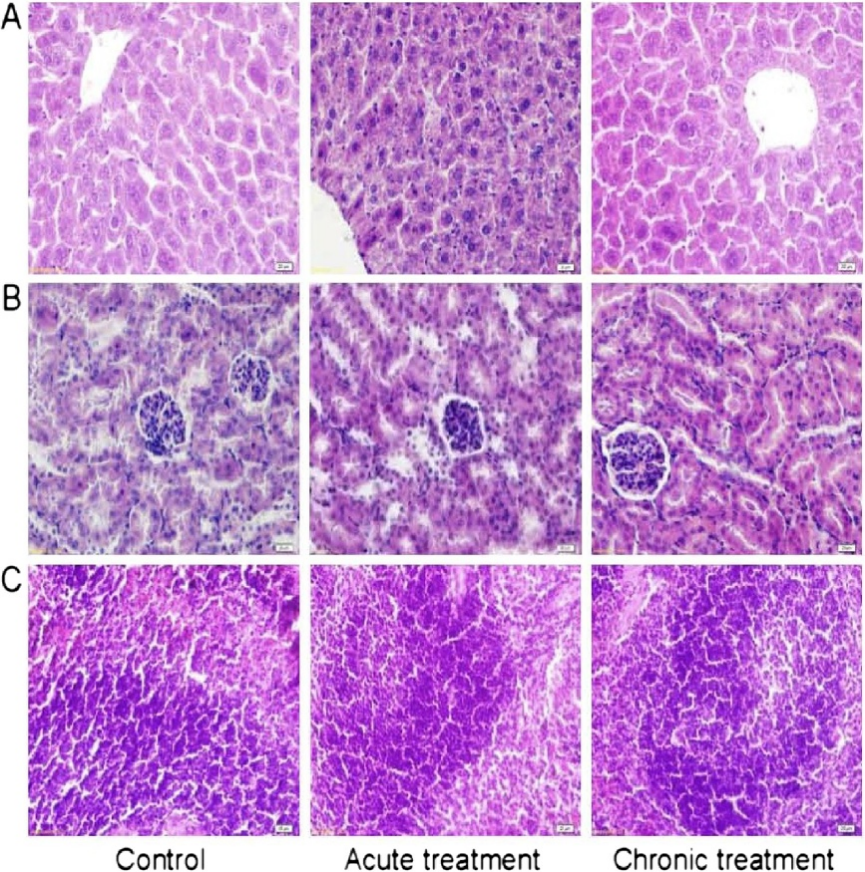
**Representative histopathological sections of the (A) liver, (B) kidney, and (C) spleen of the mice treated with HCE75 (5000 mg/kg, p.o.) once a day for 1 d (acute treatment) or 21 consecutive days (chronic treatment).** The control group was not treated. Insignificant lesions were observed in the experiments. Hematoxylin–eosin stains, 400 × original magnification.

Figure 8 shows that the histopathological examination has insignificant differences. The results reveal that HCE75 (5000 mg/kg, p.o.) administration once a day either for 1 d or 21 consecutive days does not cause lesions on the examined physiological organs. Oral HCE75 administration *per se* could thus be considered toxicologically safe.

## Conclusions

In summary, the present TST, OFT, and neurochemical analyses of the monoamine neurotransmitters in mice brain revealed the significant antidepressant effects of *H. citrina*. The effect of HCE75, the most active hydroalcoholic extract of *H. citrina*, is dose-dependent. The results confirm that such antidepressant effect is mainly related to the contributions of flavonoids, especially rutin and hesperidin. Isobologram analysis showed the sub-additive interaction between rutin and hesperidin. Toxicity and histopathological analyses confirmed that HCE75 is toxicologically safe for oral administration.
